# Genomic variation in captive deer mouse (*Peromyscus maniculatus*) populations

**DOI:** 10.1186/s12864-021-07956-w

**Published:** 2021-09-14

**Authors:** Matthew D. Lucius, Hao Ji, Diego Altomare, Robert Doran, Ben Torkian, Amanda Havighorst, Vimala Kaza, Youwen Zhang, Alexander V. Gasparian, Joseph Magagnoli, Vijay Shankar, Michael Shtutman, Hippokratis Kiaris

**Affiliations:** 1 Department of Drug Discovery and Biomedical Sciences, College of Pharmacy, University of South Carolina, Columbia, SC USA; 2 Research Computing, Division of Information Technology, University of South Carolina, Columbia, SC USA; 3 Peromyscus Genetic Stock Center, University of South Carolina, Columbia, SC USA; 4 Department of Clinical Pharmacy and Outcomes Sciences, College of Pharmacy, University of South Carolina, Columbia, SC USA; 5 Center for Human Genetics, College of Science, Clemson University, Clemson, SC USA

**Keywords:** *P. maniculatus* bairdii, *P. maniculatus* sonoriensis, Single nucleotide polymorphism, Indel, Whole genome sequencing, Genomic variation

## Abstract

**Background:**

Deer mice (genus *Peromyscus*) are the most common rodents in North America. Despite the availability of reference genomes for some species, a comprehensive database of polymorphisms, especially in those maintained as living stocks and distributed to academic investigators, is missing. In the present study we surveyed two populations of *P. maniculatus* that are maintained at the *Peromyscus* Genetic Stock Center (PGSC) for polymorphisms across their 2.5 × 10^9^ bp genome.

**Results:**

High density of variation was identified, corresponding to one SNP every 55 bp for the high altitude stock (SM2) or 207 bp for the low altitude stock (BW) using snpEff (v4.3). Indels were detected every 1157 bp for BW or 311 bp for SM2. The average Watterson estimator for the BW and SM2 populations is 248813.70388 and 869071.7671 respectively. Some differences in the distribution of missense, nonsense and silent mutations were identified between the stocks, as well as polymorphisms in genes associated with inflammation (NFATC2), hypoxia (HIF1a) and cholesterol metabolism (INSIG1) and may possess value in modeling pathology.

**Conclusions:**

This genomic resource, in combination with the availability of *P. maniculatus* from the PGSC, is expected to promote genetic and genomic studies with this animal model.

**Supplementary Information:**

The online version contains supplementary material available at 10.1186/s12864-021-07956-w.

## Introduction

Mammals of the genus *Peromyscus* (deer mice) are the most abundant rodents of North America [1–3]. Deer mice play important roles in public health as they have been identified as a natural reservoir for various infectious agents such as Hantaviruses [4, 5] and the arthropod- transmitted spirochete *B. burgdorferi* [6, 7] that cause Lyme disease, babesiosis, anaplasmosis, viral encephalitis and others. More recently *P. maniculatus* was also shown to be sensitive to SARS-CoV2 infection implying that it may also function as a secondary reservoir for the coronavirus that causes the COVID-19 pandemic [8, 9]. Because of their abundance and their characteristics, *Peromyscus* species are being used extensively as animal models for studies ranging from evolution, physiology, infectious diseases, metabolism, genetics, aging and behavior [2, 3, 10–15]. In example, *P. leucopus* lives up to 8 years in captivity as compared to the other *Peromyscus* species that reportedly live up to 4 years providing models for aging studies [16]. *P. californicus* and *P. polionotus* are strictly monogamous species that are used in studies on behavior [15, 17]. *P. eremicus* is adapted for life in the desert providing a model to explore adaptation at extreme environments [2]. *P. maniculatus* are being used for a wide array of studies ranging from metabolism and the regulation of stress response to altitude adaptation [2].

A major limitation in understanding better the impact of deer mice in public health, and in exploiting their utility as a research model is the lack of comprehensive genomic variation data in reference populations that are readily accessible to outside users [18–20]. The *Peromyscus* Genetic Stock Center at the University of South Carolina maintains different species of deer mice that are maintained as closed, genetically diverse colonies since the original caption of the original colony founders, and distributes them to outside investigators. Among them, 2 populations of *P. maniculatus* are being maintained as outbred, genetically diverse stocks: the BW stock (*Peromyscus maniculatus bairdii*) bred in captivity since 1948 and descended from 40 ancestors wild-caught near Ann Arbor, MI, and the SM2 stock (*Peromyscus maniculatus sonoriensis*) derived from about 50 animals, wild-caught by Jack Hayes in 1995 near White Mountain Research Station, CA.

Several *Peromyscus* species, have been sequenced [21, 22] while for others, including *P. maniculatus*, chromosomal assembly level reference genomes and annotations are available, providing strong foundation for genomic analyses, as opposed to scaffold-level assemblies [23–26]. Baylor College of Medicine provided a scaffold *P. maniculatus* reference genome in 2013 with a scaffold N50 of 3,760,915 and a contig N50 of 36,367 and the Hoekstra laboratory at Harvard University and HHMI provided a chromosome level *P. maniculatus* reference genome in 2018 (HU_Pman_2.1) which had a scaffold N50 of 115,033,041 and a contig N50 of 30,111 [27, 28]. Nevertheless, a genome-wide database on polymorphisms of *P. maniculatus* is lacking, restricting greatly their usage and their exploitation as genetic models. This limitation is especially pertinent to populations that are purposely maintained as outbred stocks in a stock center and are readily accessible to outside investigators. In this study we performed whole genome sequencing (WGS) to discover polymorphisms in *P. maniculatus* and initiate the establishment of a robust polymorphism database for *P. maniculatus*. Our analyses involved individuals from both the SM2 and BW populations, as an attempt to characterize these 2 distinct, yet highly relevant evolutionarily, subspecies and to record the dynamics on genomic diversity in these closed populations that are maintained in captivity for several decades.

## Materials and methods

### Samples and library preparation

Animals were sacrificed under isoflurane anesthesia. DNA samples were isolated from the liver by using the DNeasy kit (Qiagen) and quantified with the Quant-iT PicoGreen dsDNA Assay Kit (Invitrogen, Cat. No. P7589). To prepare libraries, the TruSeq DNA PCR-free Kit (Illumina, Cat. No. 20016327) was used according to manufacturer recommendations [29]. Briefly, genomic DNA (1.1 μg) was diluted to 55 μl with Resuspension Buffer. Samples were sonicated using a Covaris M220 Focused-ultrasonicator with appropriate settings to generate 350 bp fragments. Fragmented DNA was quality controlled using an Agilent 2100 Bioanalyzer and the DNA 1000 Kit (Cat. No. 5067-1504) [30]. Fragmented DNA was cleaned up with magnetic beads, end-repaired, and size selected using different ratios of the Sample Purification Beads (SPB). DNA fragments were 3′ end adenylated, TruSeq DNA Sgl index adapters (Illumina, Cat. No. 20016329 and 20016330) were ligated to the fragments’ ends, and libraries were cleaned up with SPB. Libraries were quantified by qPCR using the NEBNext Library Quant Kit for Illumina (New England Biolabs, Cat. No. E7630S) and fragment size was assessed using an Agilent 2100 Bioanalyzer and the High Sensitivity DNA Kit (Cat. No. 5067-4626) [30]. Libraries were pooled and sequenced with NovaSeq S4 (Illumina San Diego, CA) 150 bp Pair ends, by Psomagen (Rockville, MD, USA).

### SNP and Indel calling

The following pipeline used was adapted from the Genome Analysis Toolkit (GATK) Best Practices [31]. Paired-end FASTQ files of each sample were aligned to the HU_Pman_2.1 *P. maniculatus* reference genome (GCA_003704035.1, Ensembl release-96) using BWA-MEM (v0.7.17-r1188) [27, 32]. The resulting SAM files were sorted by coordinate into BAM file format using Picard (v2.18.15; http://broadinstitute.github.io/picard/) [33]. Alignment metrics and duplicate metrics are in Supplemental Tables. Using GATK (v4.0.5.1) HaplotypeCaller variants were called with default parameters and then SNPs and Indels were selected using SelectVariants default parameters. SNPs and Indels were filtered using GATK VariantFiltration with the “filter-expression” parameter with the following limits for SNPs and Indels respectively: “(QD < 2.0) || (FS > 60.0) || (MQ < 40.0) || (MQRankSum < -12.5) || (ReadPosRankSum < -8.0) || (SOR > 3.0)”; “(QD < 2.0) || (FS > 200.0) || (ReadPosRankSum < -20.0) || (SOR > 10.0)”. The GATK BaseRecalibrator tool was run and applied with the filtered SNPs and Indels followed by a repeat of variant calling and filtration with the newly recalibrated bam files. SNPs and Indels are then annotated using snpEff (v4.3); a local snpEff database was built for *P. maniculatus* using the HU_Pman_2.1.96 annotation in GTF file format [34]. The resulting variants can be accessed on the European Variation Archive (EVA) with the accession ID PRJEB41333.

### ANGSD

The thetas for BW and SM2 thetas were calculated using ANGSD (v0.930) [35]. The methods used to calculate the thetas were done according to ANGSD’s website page on Thetas, Tajima, and Neutrality tests (http://www.popgen.dk/angsd/index.php/Thetas,Tajima,Neutrality_tests) [36, 37]. The SFS estimation was calculated using 500,000,000 bp buckets. The average of the SFS estimations was then calculated across all buckets and used for the calculation of the theta values.

### Validation of SNPs

INSIG1 and HIF1a SNPs were validated via PCR and sanger sequencing of the fragments.. Gene-specific primers were ordered from Integrated DNA Technologies (Coralville, Iowa. The primer sequences for INSIG1 are as follows:

- Forward Primer: AA-TAATACGACTCACTATAGGG-TTGCCAATAATGTCCAACTG
- Reverse Primer: AA-CAGGAAACAGCTATGAC-GAGTGATCAGCGTAGCTAGG

The primer sequences for HIF1a are as follows:

- Forward Primer: AA-TAATACGACTCACTATAGGG-TGCCACCACCACCACTACTG
- Reverse Primer: AA-CAGGAAACAGCTATGAC-GGCTTTTGCGAGTTTGTTTG

Froward primers have T7 sequence extension and reverse primers have the M13 extensions attached for the following Sanger sequencing with the corresponded T7 and M13 sequencing primers. INSIG1 primers were annealed at 66 °C and HIF1a primers were annealed at 72 °C. Samples were gel-purified gel purification kit (Zymo Research, Irvine, CA) followed by Sanger sequencing.

## Results

### Overall diversity in SM2 and BW stocks

**Fig. 1:**
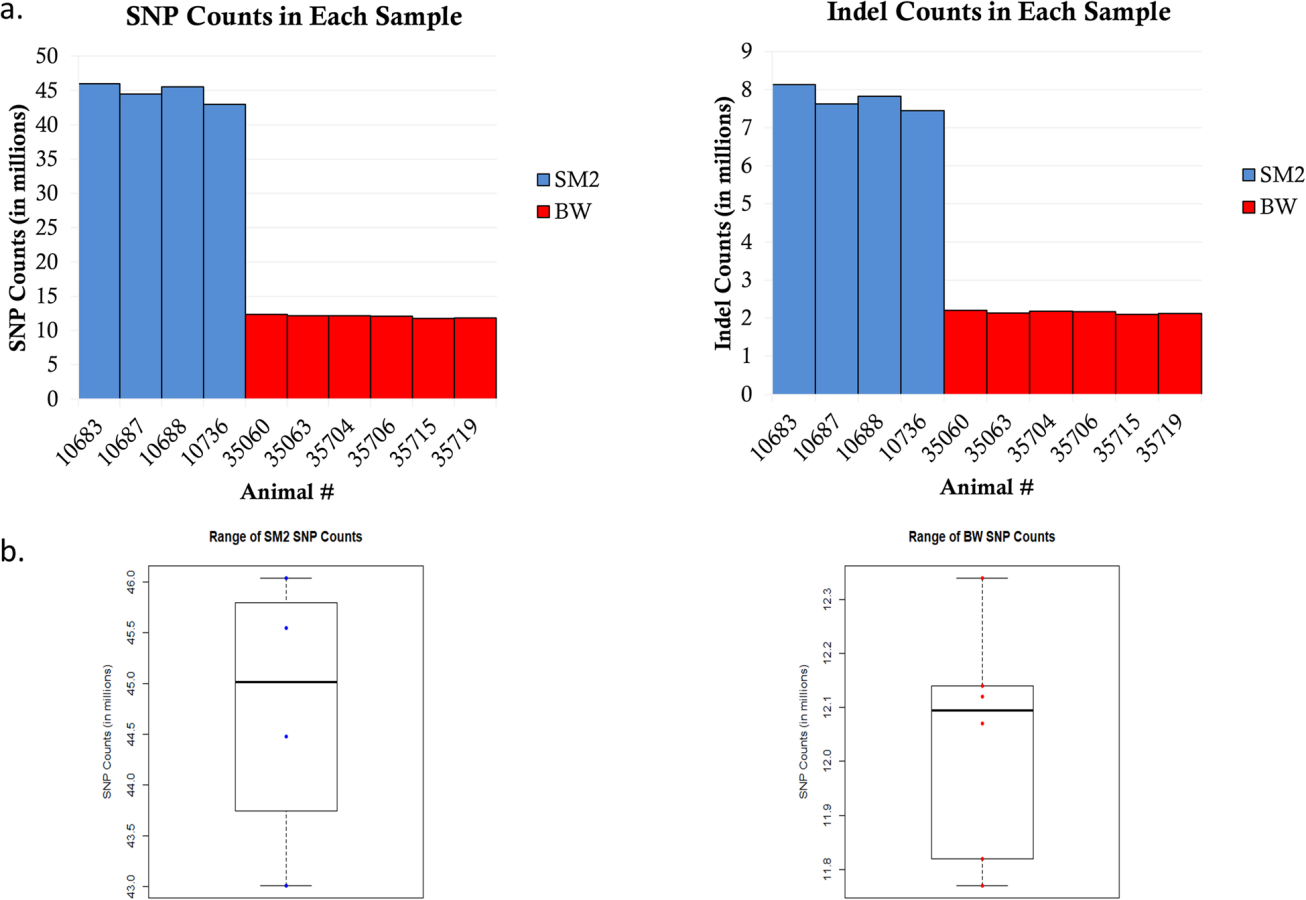
Comparison of SNP and Indel counts found in BW and SM2 Counts of SNPs and Indels (**a**) were taken for each sample. Each sample is listed in the order of their ID at the PGSC. Each count is measured in millions. SM2 samples are marked by the color blue while BW samples are marked by the color red. In (**b**) the SNP counts of SM2 and BW are organized into boxplots to compare the range of SNP counts for both SM2 and BW. The counts are measured in millions where SM2 SNP counts have a range of 3 million and BW SNP counts have a range of 0.5 million

**Fig. 2:**
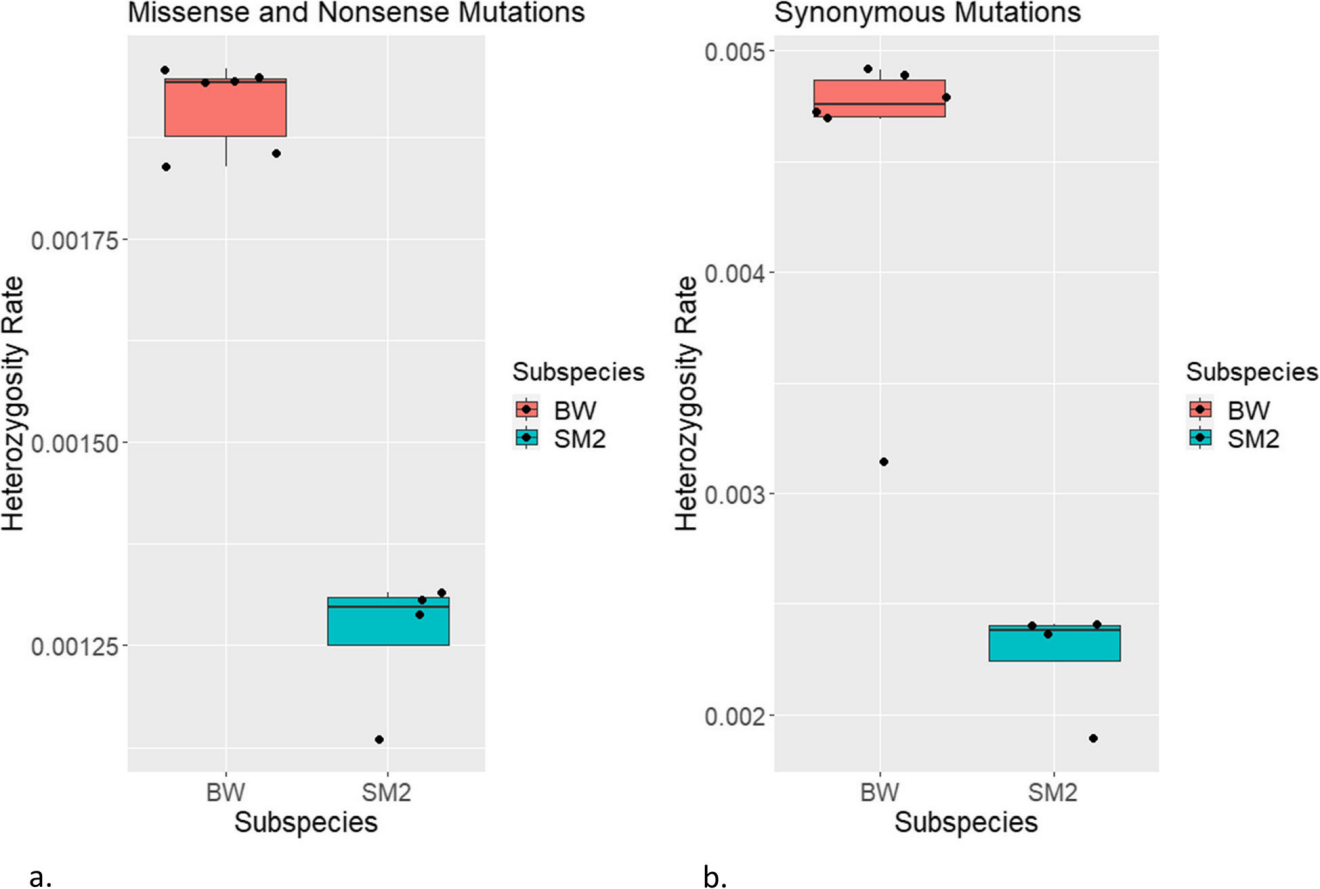
Heterozygosity of *Peromyscus maniculatus*. BW show higher heterozygosity than SM2. The missense and nonsense polymorphisms (**a**) have an average heterozygosity of 0.0013 with a standard deviation of 8.48 × 10^− 5^ in SM2 and an average heterozygosity of 0.0019 with a standard deviation of 5.29 × 10^− 5^ in BW. The synonymous polymorphisms have a higher heterozygosity than the missense and nonsense polymorphisms. The synonymous polymorphisms (**b**) have an average heterozygosity of 0.0023 with a standard deviation of 0.0003 in SM2 and an average heterozygosity of 0.0045 with a standard deviation of 0.0007 in BW

Ten (10) individuals from two *P. maniculatus* stocks were subjected to whole genome sequencing (WGS), *Peromyscus maniculatus sonoriensis* (SM2) and *Peromyscus maniculatus bairdii* (BW). The SM2 stock was established by animals captured near the White Mountain Research Station, CA in 1995 and the BW population was captured near Ann Arbor, Michigan in 1948 [2]. These 2 populations were continuously maintained isolated, as different stocks, since their original acquisition by the PGSC. The HU_Pman_2.1 reference genome was established by an individual of the BW stock (https://www.ncbi.nlm.nih.gov/genome/browse/#!/eukaryotes/11397/) but provides a decent reference for the SM2 subspecies as well; the disadvantage to using this reference genome for SM2 individuals is divergent reads in SM2 samples may not map properly to the BW reference or may not be mapped altogether. An alternate SM2 reference genome will need to be established to create accurate polymorphic calls. These two populations differ by the altitudes they are found in the wild, with SM2 being found at higher altitudes and BW being found at lower altitudes [38]. Paired-end WGS analysis with an average 34X coverage depth, a standard deviation of 5.73X, a minimum of 26.98X coverage and a maximum of 48.47X coverage indicated that BW exhibited an average of about 12.1 million single nucleotide polymorphisms (SNPs) while each SM2 had 42–46 million SNPs, across their 2.5 × 10^9^ bp genome (Fig. 1a). Using chromosome 1 as a sample of coverage of the genome, each sample had an average of 92.36% coverage for all bases with more than 10 reads per base. Each sample’s chromosome 1 had a coverage range of 2.44% and had a coverage standard deviation of 1.08%. All variant data can be found on the European Variation Archive (EVA) under the project ID PRJEB41333. The BW have a range of 2.1–2.2 million insertions/deletions (indels) and the SM2 have 7.4–8.1 million indels (Fig. 1a). Each sample in Fig. 1 is indicated by the order of their ID in the PGSC. There is an average variant rate of a SNP every 55 bp and an indel every 311 bp in SM2 according to snpEff. BW have a SNP approximately every 207 bp and an indel every 1157 bp according to snpEff. Although the reference genome is aligned for *P. maniculatus bairdii* instead of *P. maniculatus sonoriensis*, the range of SNPs and indels found in SM2 is much wider than that of BW (Fig. 1b). The total amount of BW SNPs in each individual had a lower range of 11.79 million SNPs and an upper range of 12.36 million SNPs with a total count of 17.52 million SNPs for the entire sample size. The total amount of SNPs found in each individual SM2 however had a lower range of 43.01 million SNPs and upper range of 46.06 million SNPS with a total count of 48.36 SNPs in the SM2 sample size. The Watterson’s estimator of theta for each chromosome in BW and SM2 samples was found using ANGSD. The average theta of each chromosome for BW samples is 248,813.70. The minimum theta was in chromosome 13 with a value of 49,976.30 and the maximum theta was in chromosome 2 with a value of 480,638.38. The average theta of each chromosome for SM2 samples is 869,071.77. The minimum theta was in chromosome 22 with a value of 444,356.96. The maximum theta was in chromosome 1 with a value of 1,537,521.37. The missense and nonsense polymorphisms have an average heterozygosity of 0.0013 with a standard deviation of 8.48 × 10^− 5^ in SM2 and an average heterozygosity of 0.0019 with a standard deviation of 5.29 × 10^− 5^ in BW (Fig. 2a). The synonymous polymorphisms have an average heterozygosity of 0.0023 with a standard deviation of 0.0003 in SM2 and an average heterozygosity of 0.0045 with a standard deviation of 0.0007 in BW (Fig. 2b).

### Variation in the incidence of missense, nonsense and silent mutations between SM2 and BW

**Fig. 3:**
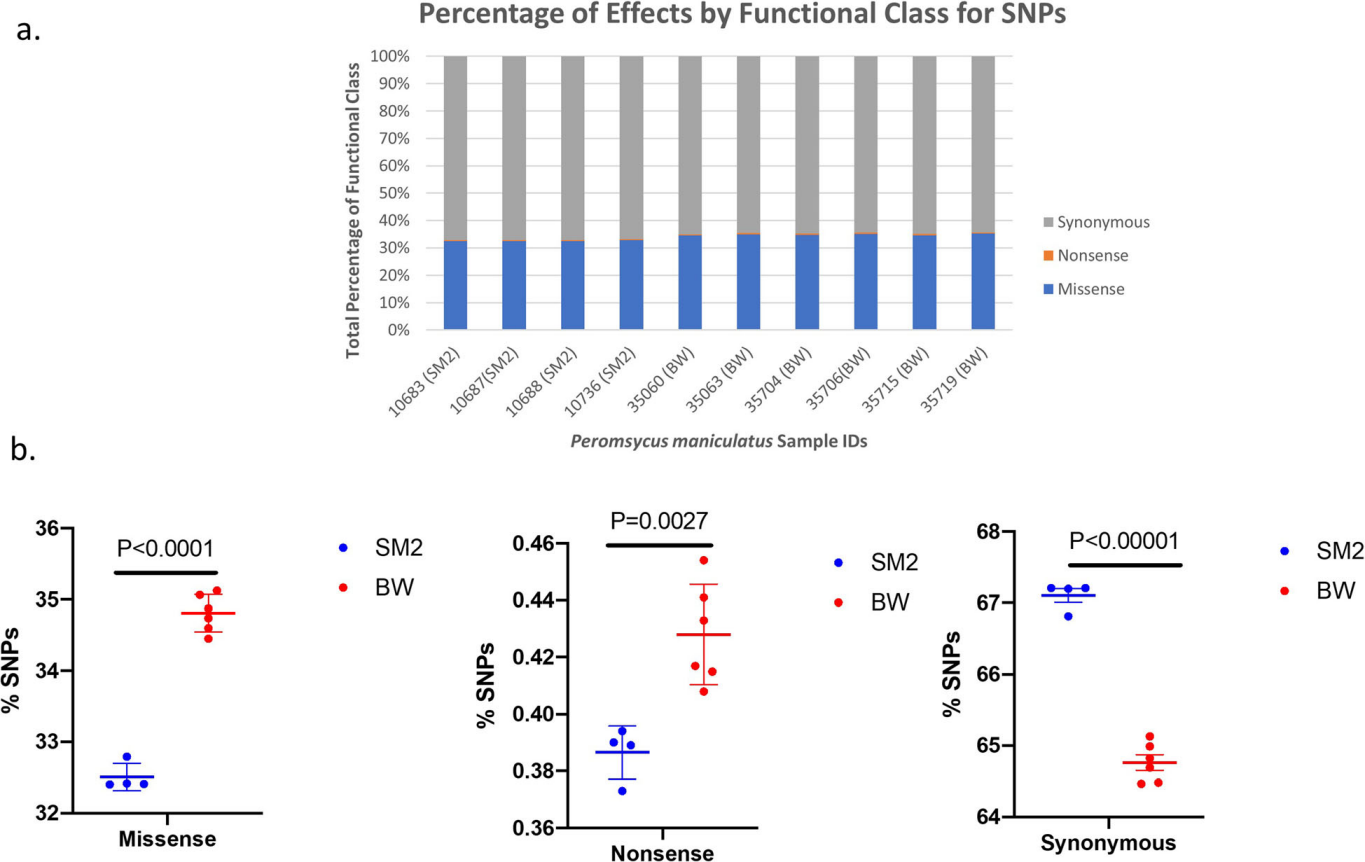
*Peromyscus maniculatus* SNP functional class. In (**a**) the percentage of the functional class of all SNPs in each sample is shown. In (**b**) the difference between the percentage of each SNP functional class for BW and SM2 samples are shown. SM2 samples are shown in blue whereas BW samples are shown in red

Between the stocks, the distribution of missense, nonsense and silent mutations exhibited differences: When expressed as a fraction of total polymorphisms identified, silent mutations prevailed in SM2 while missense and nonsense mutations in gene coding regions were significantly higher in the individuals of the BW stock (Fig. 3).

**Fig. 4:**
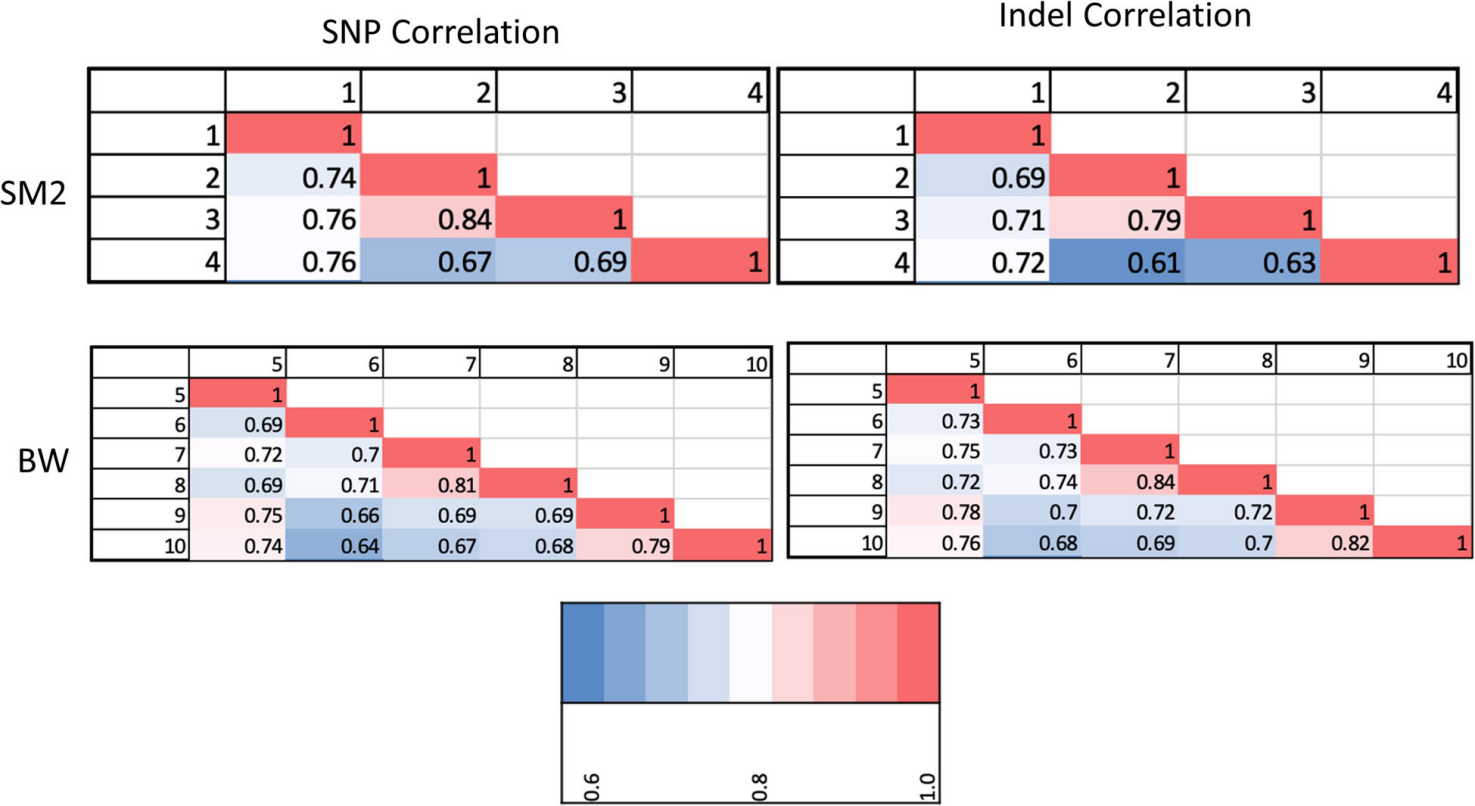
*Peromyscus maniculatus* Interspecies Polymorphism Correlation. In the upper and lower panels the correlation of polymorphisms between each SM2 samples and BW samples are shown. SNPs and Indel correlations are shown in left and right respectively. The sample numbers are found on the upper and left border of each table with the correlation percentage in the cross between two samples. The range of the correlation for each sample pair ranged from 0.6 to 0.85. Each correlation was normalized to the mean number of respective polymorphisms across all samples of the respective subspecies

The shared SNPs and indels between SM2 and BW populations have also been investigated (Fig. 4). The correlations were found by finding the number of SNPs/indels that matched between each sample pairing and normalizing to the average number of SNPs/indels for each respective subspecies. Each *P. maniculatus* pairing had a correlation between 0.6 and 0.85.

**Fig. 5:**
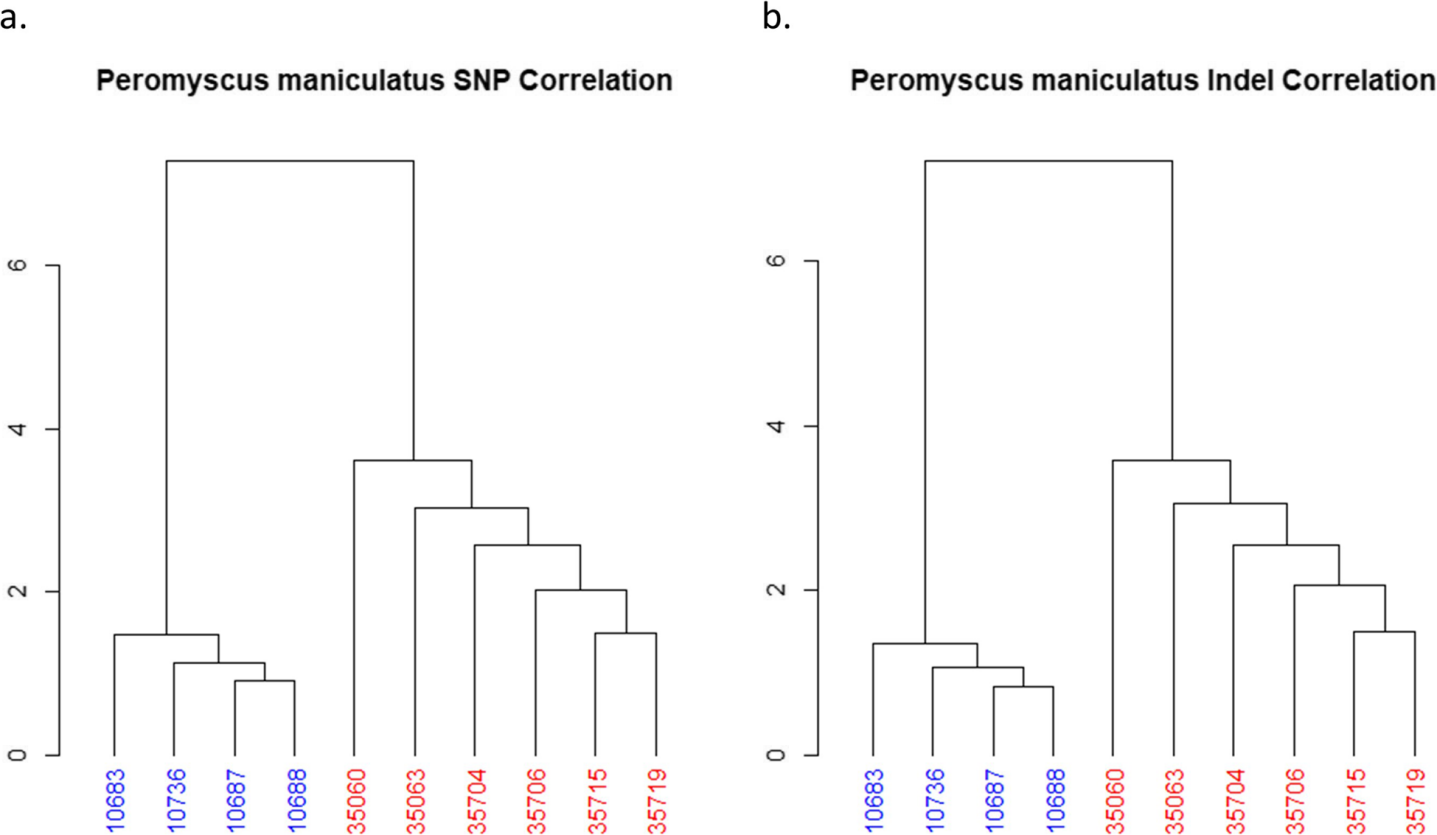
*Peromyscus maniculatus* Polymorphism Clustering. The matching polymorphisms were taken between each sample and clustered to show the relationship between the SM2 and BW *Peromyscus*. The top two clusters of each dendrogram show a separation between SM2 and BW based on both SNPs (**a**) and indels (**b**). Each sample ID is shown with their color correlating with the subspecies (BW: red, SM2: blue). The y-axis shows the relative distance between each sample

The total correlation between all samples was shown to demonstrate the relationship for both SM2 and BW (Fig. 5). In Fig. 5 dendrograms are used to shows the SM2 and BW samples are distinctly separated with clustered polymorphisms for both SNPs and indels.

### Indels, insertions and deletions in BW and SM2

**Fig. 6:**
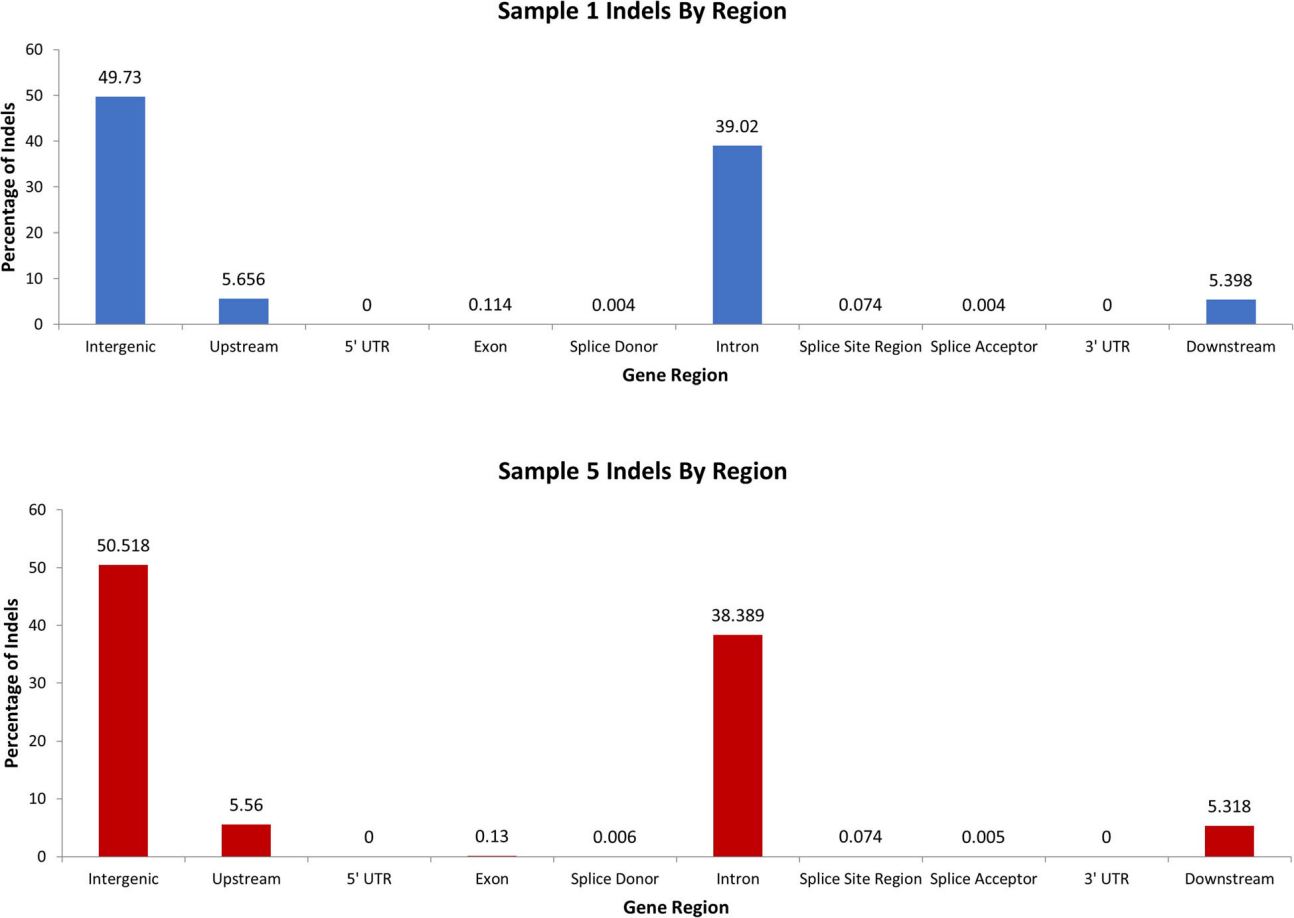
Gene regions where SM2 and BW indels are found. The percentage of the gene regions where indels were found is shown here. Even though SM2 have significantly higher counts of indels than BW, the percentage of indels found in a region are identical. The samples shown were randomly chosen to represent SM2 and BW samples. Sample 1 (10683) is an SM2 and sample 5 (35060) is a BW

**Fig. 7:**
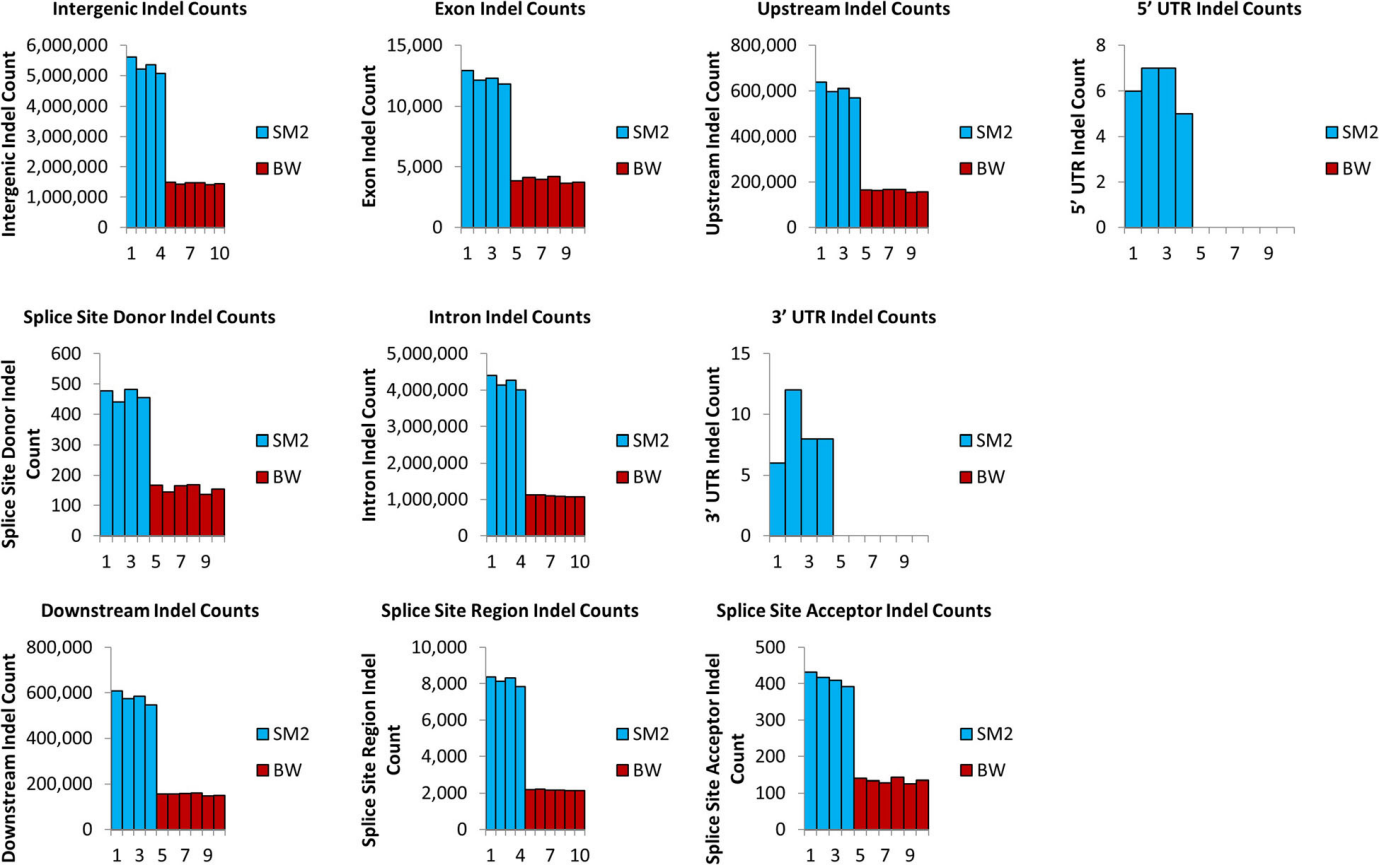
Indels found in each gene region across all samples. Each gene region is separated to show indel count in SM2 (blue) and BW (red) samples. A majority of indels are found in intergenic and intronic regions

**Fig. 8:**
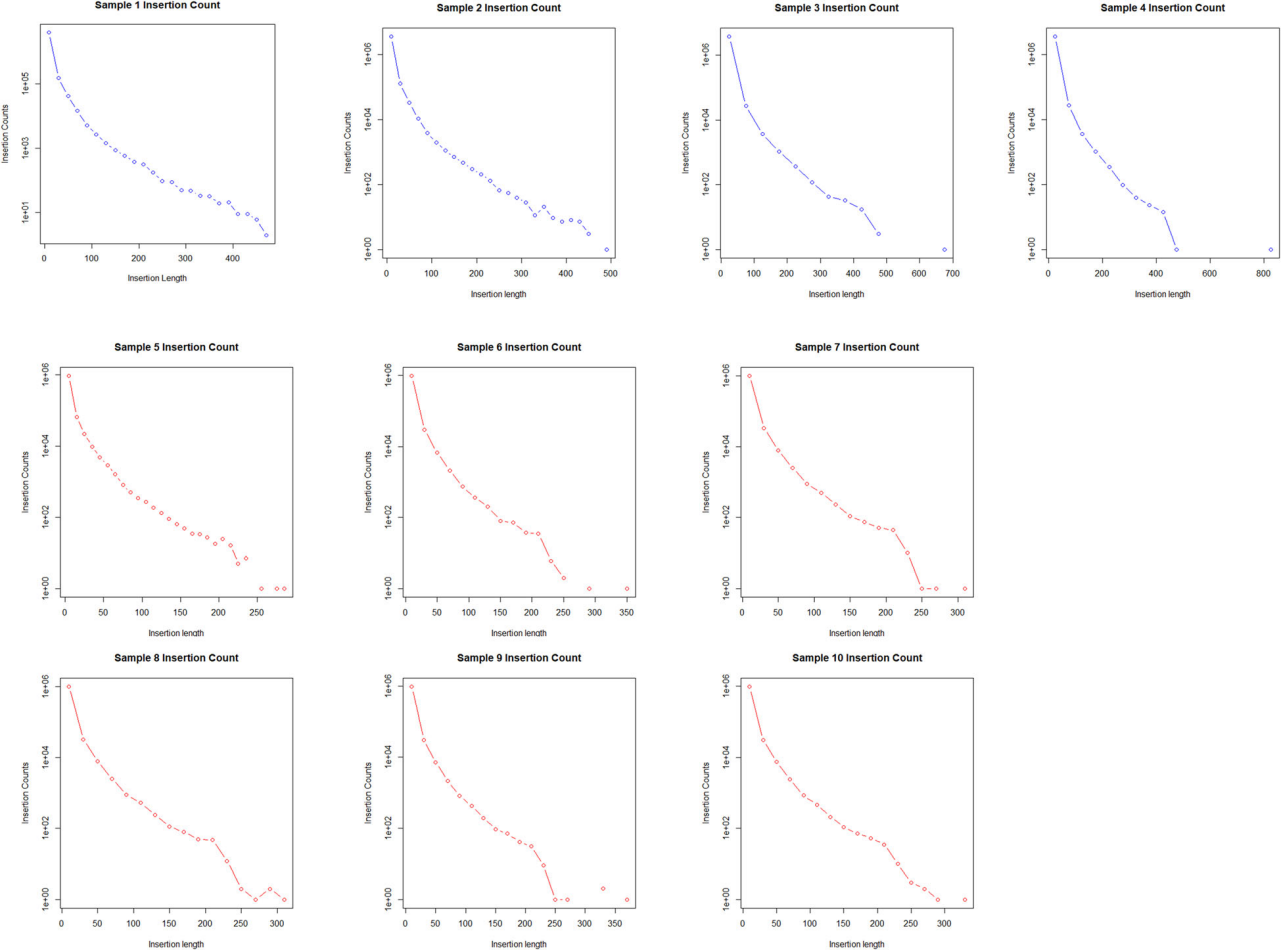
Count of insertions in all samples. The graphs here show the count of insertions in each sample as related to their length. Samples 1–4 are SM2 and samples 5–10 are BW. Insertions in the SM2 samples reach up to at least 450 bp whereas insertions in the BW samples reach up to at least 250 bp

**Fig. 9:**
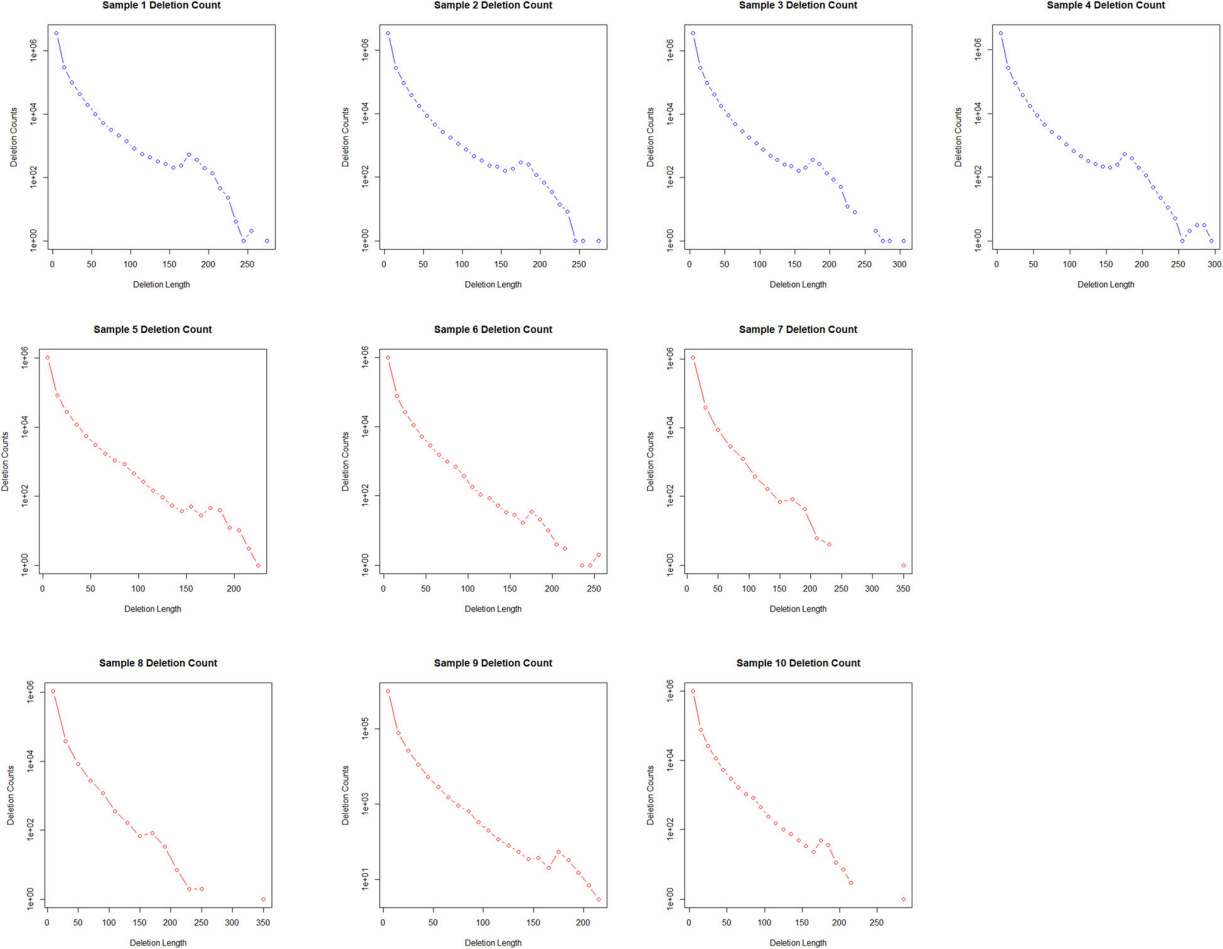
Count of deletions in all samples. The graphs here show the count of deletions in each sample as related to their length. Samples 1–4 are SM2 and samples 5–10 are BW. Deletions in the SM2 samples reach up to at least 250 bp whereas insertions in the BW samples reach up to at least 200 bp

The distribution of indels in relation to coding regions were almost identical in both stocks and was highest in the intergenic and intronic regions, followed by areas upstream and downstream of coding sequences and being minimal in 5′ and 3′ UTR, exonic sequences and splice donor and acceptor sites (Fig. 6). Surprisingly, in 5′ and 3′ UTR no indels were detected in BW and only a small number of indels were detected in SM2, despite that in exon regions indels ranged to the levels of about 12 × 10^3^ and 4 × 10^3^ per specimen in SM2 and BW respectively (Fig. 7). This is opposed to human UTR regions which contain multiple indels [39]. A number of insertions and deletions, ranging from 1 bp upwards to about 500 bp were detected in both stocks. Their distribution followed in both SM2 and BW, an exponentially declininag pattern and especially the deletions, exhibited a transient peak, at around 180 bp corresponding to 100 incidences per specimen (Figs. 8 and 9).

### Occurrence of SNPs and Indels across individual chromosomes

A quick glance at each individual chromosome shows there are differing variant rates in each chromosome. A random sample was taken for BW and SM2 and the variant rate in each chromosome was calculated based off the number of variant occurrences and the length of the chromosome (Supplementary Tables 1, 2). SNPs had a quicker variant rate than Indels throughout all respective chromosomes. For SNPs, sample 8 (35706) had a lower bound variant rate of 1 variant every 880 base pairs in chromosome 13 and an upper bound variant rate of 1 variant every 116 base pairs in chromosome 17. The indel variant rate in sample 8 had a lower bound of 1 variant every 4309 base pairs in chromosome 13 and an upper bound of 1 variant every 661 base pairs in chromosome 17. The SM2 SNPs in sample 4 (10736) had a lower bound variant rate of 1 variant per 40 base pairs in chromosome 18 and an upper bound variant rate of 1 variant per 103 base pairs in the X chromosome. The indel variant rates in sample 4 ranged from 1 variant per 221 base pairs in chromosome 18 to 1 variant per 513 base pairs in the X chromosome.

**Fig. 10:**
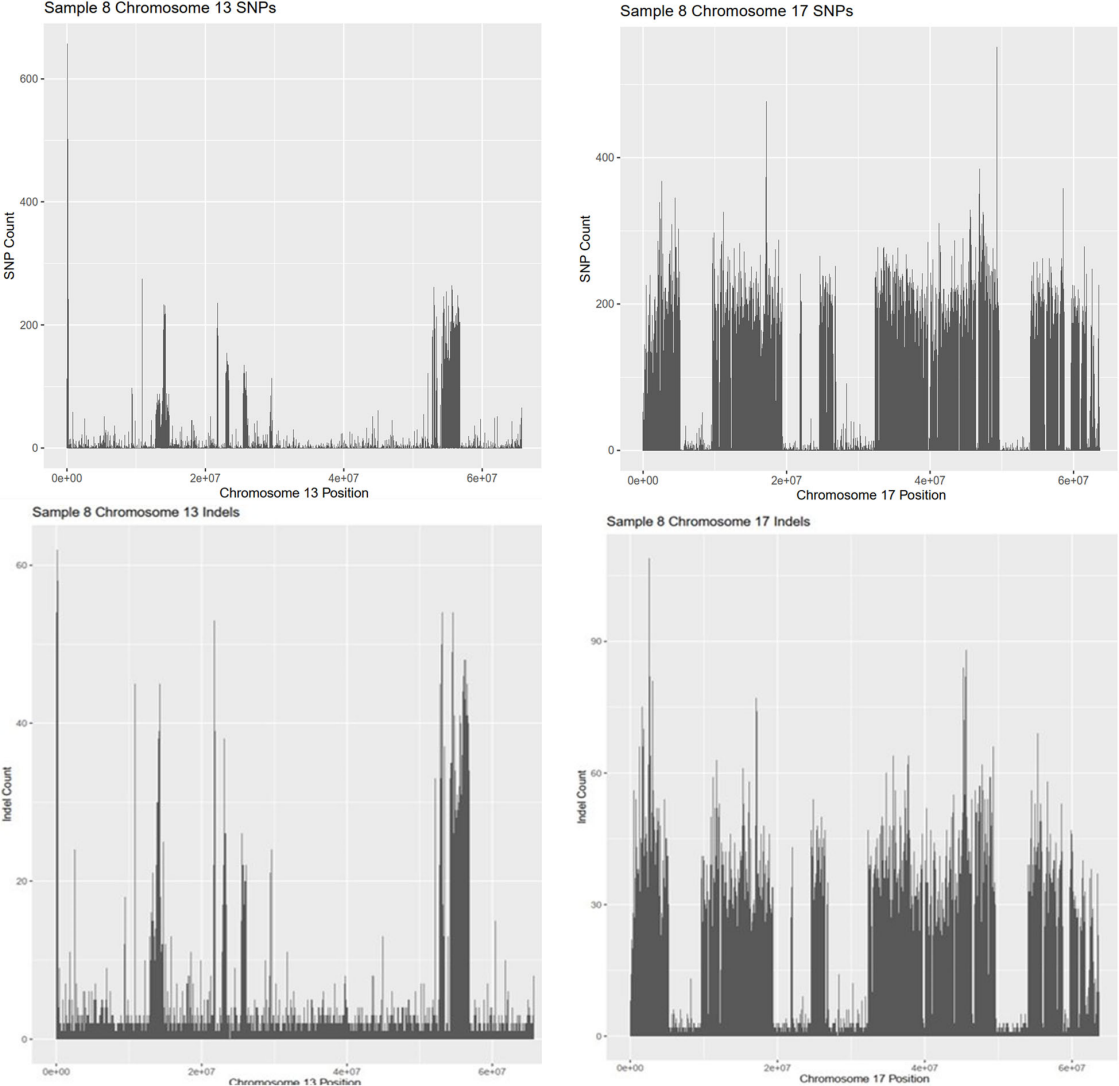
Variant Coverage in a BW Peromyscus. Sample 8 (35706) was used as a random example BW to show the range of coverage of SNPs and Indels in an individual chromosome. Chromosome 13 had the least amount of SNPs and Indels (Left) whereas chromosome 17 had the greatest amount of SNPs and Indels (Right). Variants were counted in bins of 10,000 bp

**Fig. 11:**
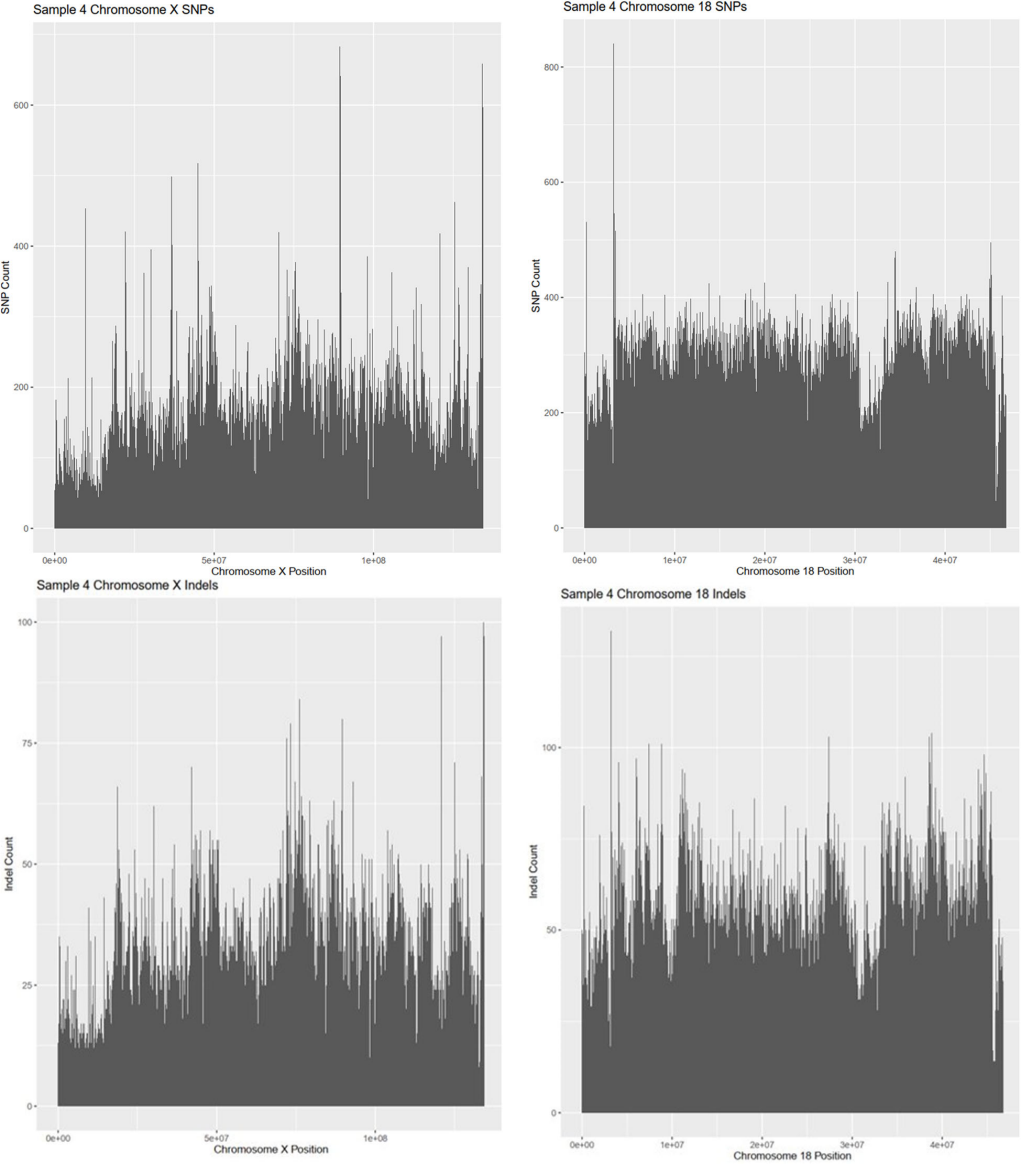
Variant Coverage in an SM2 Peromyscus. Sample 4 (35706) was used as a random example SM2 to show the upper and lower bounds of coverage of SNPs and Indels in an individual chromosome for SM2 Peromyscus. The X chromosome had the least amount of SNPs and Indels (Left) whereas chromosome 18 had the greatest amount of SNPs and Indels (Right). Variants were counted in bins of 10,000 bp

Coverage of the genome for SNPs and indels was also shown by the SNPs and indels in chromosomes with the least and the greatest number of polymorphisms for each subspecies. BW samples were represented by sample 8 (Fig. 10) and SM2 samples were represented by sample 4 (Fig. 11). The peaks shown in Figs. 10 and 11 are the number of polymorphisms found in 10,000 base pair bins. SM2 samples show greater coverage than BW samples due to greater numbers of polymorphisms.

### Missense and nonsense mutations of potential biomedical value

Of note is a roster of specific mutations that were identified indicating the existence of polymorphisms in disease-associated genes. Some of them were seen only in one stock while others in both stocks were assessed.

#### Missense mutations

Many of the polymorphisms found in *P. maniculatus* created missense mutations. Some of the representative missense mutations found were in the NFATC2, and HIF1α genes. NFATC2 (nuclear factor of activated T cells) 2, is part of the T cells transcription complex which plays a role in gene transcription during an immune response [40, 41]. NFATC2 contained a missense mutation which caused a threonine to alanine substitution (T133A). This predicted change from a non-polar amino acid to a polar amino acid may cause substantial changes in NFATC2 conformation [42]. This missense mutation was found in 3 out of 4 SM2 samples and 2 out of 6 BW samples. Two of the SM2 are homozygous whereas all other samples with this mutation were heterozygous.

HIF1α, hypoxia induced factor 1 alpha subunit, is part of a heterodimeric structure which takes role during the hypoxia response [43]. There are two missense mutations within the HIF1α gene, S630A and V662I. These polymorphisms were only seen in SM2 stock, with an allelic frequency of about 0.75, and were validated in 20 additional individuals per stock. Given the role of HIF1a in regulating the response to hypoxia it is plausible that these polymorphisms, if they existed in the original founders of the colony, are related to the high-altitude adaptation of the SM2 animals.

#### Stop codons

Along with missense mutations found in *P. maniculatus*, there were also nonsense mutations leading to stop codons causing premature termination of translation. These nonsense mutations could be used to create natural knockout models in the context of a naturally existing wild type population. A few representative nonsense mutations found were in INSIG1, POLQ, and LRP5.

INSIG1, also known as insulin induced gene 1, is an ER protein responsible for regulating cholesterol metabolism, lipogenesis, and glucose homeostasis, mainly through the binding of SREBP cleavage-activating protein (SCAP) [40, 44]. The protein is a transmembrane 259 amino acids long with the mutation creating a stop codon on amino acid 190 (R190*). The active site of INSIG1 is Aspartic Acid 187 on the end of the 4th transmembrane domain [45, 46]. Although the active site of INSIG1 is before the nonsense mutation, this still creates a truncated protein and changes the conformation. Despite the high prevalence of the mutant allele in our stocks, no homozygous animals were identified implying lethality. This was confirmed in assaying 37 randomly selected *P. maniculatus* individuals in which allelic frequencies exhibited significant deviation from Hardy-Weinberg equilibrium using Fisher’s exact test (*P* = 0.045). Thirteen *P. maniculatus* had a homozygous wildtype genotype whereas 24 *P. maniculatus* had a heterozygous genotype and no *P. maniculatus* had a homozygous mutant genotype. Furthermore, breeding of heterozygous animals failed to produce homozygous mutant offspring.

POLQ is the gene for DNA Polymerase Theta, a polymerase necessary for microhomology-mediated end joining (MMEJ) [40, 47]. A stop codon was identified at amino acid 330 (R330*) out of 2550 amino acids. This was seen in 2 heterozygous SM2 and appeared homozygous wildtype in all other samples.

LRP5, low-density lipoprotein receptor-related protein 5, plays a role in affecting bone mass accrual during development and skeletal homeostasis [40, 48]. A stop codon was identified at E273*. This was only seen in SM2 *Peromyscus*, all of which were heterozygous, and homozygous wildtype in all BW *Peromyscus* samples.

## Discussion

In the present study we report a comprehensive roster of polymorphisms detected in two populations of *P. maniculatus* that are maintained in the PGSC. The analysis covered 2.5X10^9^ bp of the *P. maniculatus* genome and revealed the presence of about 17.5X10^6^ SNPs and 2.1X10^6^ indels for BW stock, against the publicly available genome assembly. Variation was about 4 times higher in the animals of the SM2 stock. This high density of polymorphisms, especially if individuals of the two stocks interbreed, provides tremendous genetic power in mapping loci of interest.

The higher polymorphism count in SM2 is likely due to the genetic divergence of the two populations and the fact that SM2 samples were aligned to the BW genome, due to a lack of an SM2 reference genome. Nevertheless, the fact that the range of variation within animals of the same stock was higher for the SM2, despite only 4 SM2 as opposed to 6 BW individuals were sequenced, may suggest that SM2 stock could have higher allelic diversity than BW, but a larger sample size would be needed to support this argument. Heterozygosity was lower though in the SM2, implying lower intrapopulation diversity as compared to BW which can be due to the different history of the stocks in our facilities: Both stocks were originally established by similar methods, 40–50 wild caught animals and this discrepancy between BW and SM2 stocks may reflect the diversity of the original founders. In addition, BW are utilized at a higher degree than the SM2 and the BW colony was established about 40 years earlier. Therefore BW are more actively bred at the PGSC which may occasionally result in a series of bottlenecks that compromises their diversity as recorded today. Regardless of SM2 having higher occurrences of variants than BW, both BW and SM2 have full coverage of variants across each chromosome. These rates of genetic variation are comparable in magnitude to those reported for conventional laboratory mice (Mus) at which 71 X 10^6^ SNPs and 12 X 10^6^ M indels have been identified across 13 inbred mouse strains [49].

In comparing the distribution of the polymorphisms in coding regions between the stocks, a noteworthy observation was made, related to the bias seen in SM2 for the type of mutations identified: In the SM2 stock, the fraction of missense and nonsense polymorphisms expressed as a proportion of the total SNPs identified, was lower than that of BW, while synonymous polymorphisms were more common in SM2. The small population size, the differences in the breeding programs and history of the two stocks in captivity due to differences in the demand of the stocks, and the original extraction of variation data by alignment of the SM2 individuals to BW genome, highly restricts the extraction of evolutionarily relevant conclusions.

In addition to its value in characterizing the genomic architecture of these commonly used stocks of *P. maniculatus*, the present analyses also revealed a roster of loss of function alleles and mutant alleles that could be used to generate animals with desired genotypes in the context of a natural population. Those included in example a truncated form of the cholesterol biosynthesis regulator INSIG1, and HIF1a polymorphisms. In the case of INSIG1 it has been shown that INSIG1/INSIG2 knockouts in *Mus musculus* still lead to viable offspring that produce much higher levels of cholesterol as a result [50]. In *P. maniculatus* though, truncation of INSIG1 in homozygosity apparently leads to lethality. Whether this is related to *Peromyscus* physiology, or toxicity of the truncated INSIG1 allele remains to be established. The high frequency though of this allele in heterozygosity implies some advantages in the individuals that cause its stabilization in the population. HIF1a variation may also be of special value since they were seen, in high frequency in the SM2 animals only. Whether this polymorphism possesses evolutionary relevance remains to be established since the animals were bred for several generations in captivity and this SNP may be a de novo mutation that emerged in our colony.

Besides its value in describing the landscape of diversity in captive *Peromyscus* stocks and in pointing to specific, functional polymorphisms of potential biological value, this resource has an additional significance: All individual animals at the PGSC, including those supplied to investigators worldwide, are pedigreed and can be traced back to their original ancestors. Thus, this resource provides abundant baseline genetic data that refer to a reference, genetically diverse population. This in turn greatly facilitates both retrospective analyses of specimens derived in the past and studies that can be implemented in the future using animals with comparable genetic make-up. Finally, even though the genetic variation data reported here do not accurately reflect the variation of *P. maniculatus* in the wild, the present results may be of use to investigators addressing the genomic diversity of wild-caught *P. maniculatus*.

## Supplementary Information

**Additional file 1 **: **Supplementary Table 1.** Variant Rates of *Peromyscus maniculatus* bairdii. **Supplementary Table 2.** Variant Rates of *Peromyscus maniculatus* sonoriensis.

## Data Availability

Animals are available from the *Peromyscus* Genetic Stock Center located at the University of South Carolina. DNA sequences have been deposited to the European Variation Archive (EVA) under the project ID PRJEB41333 (https://www.ebi.ac.uk/ena/browser/view/PRJEB41333?show=analyses). Public access is open for PRJEB41333.
