# Supplementary material for: Genomic variation in captive deer mouse (Peromyscus maniculatus) populations
Source: BMC Genomics. 2021 Sep 14;22:662. doi: 10.1186/s12864-021-07956-w (PMC8438655; doi:10.1186/s12864-021-07956-w)
Supplement: Supplementary file 1 — Additional file 1 : Supplementary Table 1. Variant Rates of Peromyscus maniculatus bairdii. Supplementary Table 2. Variant Rates of Peromyscus maniculatus sonoriensis. [file 12864_2021_7956_MOESM1_ESM.pdf]

Genomic variation in captive deer mouse (*Peromyscus maniculatus*) populations

Matthew D. Lucius[1], Hao Ji [1], Diego Altomare [1], Robert Doran[2], Ben Torkian[2], Amanda Havighorst[1], Vimala Kaza[3], Youwen Zhang[1], Alexander V. Gasparian[1], Joseph Maganoli[4], Vijay Shankar[5], Michael Shtutman[1], and Hippokratis Kiaris[1,3]*

[1]Department of Drug Discovery and Biomedical Sciences, College of Pharmacy, University of South Carolina, SC, USA.

[2]Research Computing, Division of Information Technology, University of South Carolina, SC, USA

[3]*Peromyscus* Genetic Stock Center, University of South Carolina, SC, USA

[4]Department of Clinical Pharmacy and Outcomes Sciences, College of Pharmacy, University of South Carolina, Columbia, SC, USA

[5]Center for Human Genetics, College of Science, Clemson University, SC, USA

***, Correspondence**: H. Kiaris (hk@sc.edu)

Supplementary Table 1

Sample 8 Variant Rate (35706)			
Chromosome	Chromosome Length	SNP Rate	Indel Rate
1	193,310,054	212	1,198
2	168,715,211	129	707
3	161,151,335	264	1,444
4	154,712,973	186	987
5	139,359,418	246	1,330
6	134,808,455	293	1,651
7	119,402,526	247	1,281
8	108,536,456	186	944
9	115,033,041	209	1,136
10	98,965,349	188	1,045
11	94,517,625	239	1,335
12	83,173,863	319	1,675
13	65,685,024	880	4,039
14	88,525,187	440	2,219
15	78,974,444	196	1,081
16	63,928,082	257	1,403
17	63,635,831	116	661
18	46,762,208	169	937
19	79,940,924	140	799
20	70,296,724	210	1,155
21	70,257,074	137	749
22	54,709,200	197	1,013
23	47,673,962	232	1,246
X	134,369,076	543	2,636

Supplementary Table 1 Variant Rates of Peromyscus maniculatus bairdii Sample 8 was taken as a random representative example of BW Peromyscus. The SNP and Indel rates were made by taking the total count of SNPs/Indels in a chromosome and dividing it across the length of each respective chromosome. The SNP rates are much greater than the Indel rates for each chromosome.

Supplementary Table 2

Sample 4 Variant Rate (10736)			
Chromosome	Chromosome Length	SNP Rate	Indel Rate
1	193,310,054	61	356
2	168,715,211	58	319
3	161,151,335	55	314
4	154,712,973	68	385
5	139,359,418	60	334
6	134,808,455	56	327
7	119,402,526	70	394
8	108,536,456	65	337
9	115,033,041	54	312
10	98,965,349	50	280
11	94,517,625	51	295
12	83,173,863	46	257
13	65,685,024	57	322
14	88,525,187	73	419
15	78,974,444	53	310
16	63,928,082	49	279
17	63,635,831	49	291
18	46,762,208	40	221
19	79,940,924	51	299
20	70,296,724	51	278
21	70,257,074	55	301
22	54,709,200	50	275
23	47,673,962	50	263
X	134,369,076	103	513

Supplementary Table 2 Variant Rates of Peromyscus maniculatus sonoriensis Sample 4 was taken as a random representative example of SM2 Peromyscus. The SNP and Indel rates were made by taking the total count of SNPs/Indels in a chromosome and dividing it across the length of each respective chromosome. The sample was aligned to the reference genome for Peromyscus maniculatus bairdii. The SNP rates are much greater than the Indel rates for each chromosome. SM2 variant rates are much greater than BW variant rates.
